# Diabetes, undernutrition, migration and indigenous communities: tuberculosis in Chiapas, Mexico

**DOI:** 10.1017/S0950268818003461

**Published:** 2019-01-25

**Authors:** H. A. Rashak, H. J. Sánchez-Pérez, B. E. Abdelbary, A. Bencomo-Alerm, N. Enriquez-Ríos, A. Gómez-Velasco, A. Colorado, M. Castellanos-Joya, M. H. Rahbar, B. I. Restrepo

**Affiliations:** 1 University of Texas Health Houston, School of Public Health, Brownsville Campus, Brownsville, TX, 78520, USA; 2 Research Network GRAAL (Research Groups for Africa and Latin America), The College of the South Border (ECOSUR), San Cristobal de Las Casas Chiapas, Mexico; 3 Department of Physician Assistant, University of Texas Rio Grande Valley, College of Health Affairs, Edinburg campus, Edinburg, TX, 78541, USA; 4 GRAAL, Prevention and Control Program of Tuberculosis in the Highlands Region of Chiapas, Ministry of Health of Chiapas, Chiapas, Mexico; 5 Transmissible and Non-transmissible Diseases Department, Ministry of Health of Chiapas, Chiapas, Mexico; 6 Advocate and International Public Health Consultant Fighting Tuberculosis, HIV/AIDS and other Neglected Diseases, San Diego, California, USA; 7 Ministry of Health, Mexico City, Mexico; 8 Department of Epidemiology, Human Genetics and Environmental Sciences, School of Public Health, University of Texas Health Science Centre at Houston, Houston, Texas, USA; 9 Division of Clinical and Translational Sciences, Department of Internal Medicine, McGovern Medical School, and The Centre for Clinical and Translational Sciences, University of Texas Health Science Centre at Houston, Houston, Texas, USA

**Keywords:** Diabetes mellitus (DM), indigenous, Mexico, tuberculosis (TB), undernutrition

## Abstract

We investigated the distribution of comorbidities among adult tuberculosis (TB) patients in Chiapas, the poorest Mexican state, with a high presence of indigenous population, and a corridor for migrants from Latin America. Secondary analysis on 5508 new adult TB patients diagnosed between 2010 and 2014 revealed that the most prevalent comorbidities were diabetes mellitus (DM; 19.1%) and undernutrition (14.4%). The prevalence of DM in these TB patients was significantly higher among middle aged (41–64 years) compared with older adults (⩾65 years) (38.6% *vs.* 23.2%; *P* < 0.0001). The prevalence of undernutrition was lower among those with DM, and higher in communities with high indigenous presence. Immigrants only comprised 2% of all TB cases, but were more likely to have unfavourable TB treatment outcomes (treatment failure, death and default) when compared with those born in Chiapas (29.5% *vs.* 11.1%; *P* < 0.05). Unfavourable TB outcomes were also more prevalent among the TB patients with undernutrition, HIV or older age, but not DM (*P* < 0.05). Our study in Chiapas illustrates the challenges of other regions worldwide where social (e.g. indigenous origin, poverty, migration) and host factors (DM, undernutrition, HIV, older age) are associated with TB. Further understanding of these critical factors will guide local policy makers and health providers to improve TB management.

## Introduction

Tuberculosis (TB) remains a major health challenge worldwide, affecting 10.4 million people and killing 1.3 million HIV-negative people in 2016 [1]. It is the leading cause of death from an infectious agent, ranking above HIV/AIDS. Low- and middle-income countries account for 97% of the reported TB cases globally. Mexico is a middle-income country where TB is a major public health issue, with an incidence rate of 22 per 100 000 (28 000 cases) and a mortality rate of 2.3 per 100 000 (~3000 deaths) in 2016 [1]. Within Mexico, there is variability in the morbidity and mortality attributed to TB, with the highest rates in the poorest states [2]. Our study focuses on the state of Chiapas, which is the poorest in Mexico with 77.1% of its population living under poverty conditions [3]. It is ranked 11th in TB incidence with 24/1 00 000 cases in 2016, but this statistic is likely higher due to the under-reporting of TB in this state [4]. Furthermore, TB mortality rates are twice as high in Chiapas when compared with Mexico as a whole, and Chiapas is ranked 6th in the number of multi-drug-resistant (MDR)-TB cases [4–6].

The most important risk factors for TB in Mexico are diabetes mellitus (DM), under-nutrition, HIV/AIDS and alcoholism [2, 7]. DM is the most prevalent in TB patients, with some regions of Mexico having among the highest estimates worldwide [7–9]. In a study representing adults 20 years or older in the entire Mexican territory, the average prevalence of DM among TB patients was 19.3% among 181 378 TB patients reported to the health authorities between 2000 and 2012 [9]. This average resulted from large fluctuations that depend on the region of Mexico and on active *vs.* passive reporting. For example, in northern Tamaulipas the prevalence of DM by passive reporting was 18% between 1998 and 2004, and in 2006–2008 the prevalence was 36% in a research study using systematic evaluation of hyperglycaemia and glycated haemoglobin [7]. In TB patients, DM has also been associated with worse TB treatment outcomes *vs.* no DM. These include delayed clearance of mycobacteria, treatment failure and death [9–16]. However, some studies do not find such differences by DM status, and some even report more favourable TB treatment outcomes for TB-DM patients [17, 18]. In Chiapas, in 2017 the prevalence of DM was 9.7% in the general population in 2017 [19], and we estimated it 28.7% among TB patients (Sanchez-Perez, unpublished observation). However, further data on the epidemiology of TB and DM in Chiapas is scanty, including the contribution of DM to TB outcomes.

Undernutrition (or low body mass index; BMI) and TB have a bidirectional relationship. The immunodeficiency caused by undernutrition increases the risk of TB, and TB can cause further undernutrition due to its increased metabolic demands and decreased appetite [20]. The World Health Organization (WHO) estimates that undernutrition causes about one quarter of all new TB cases globally, and they have published guidelines on the nutritional care of TB patients [21]. Nutritional deficiencies also delay TB recovery, and increase the risk of death or of relapse [22, 23]. In 2012, the prevalence of stunting in children ⩽5 years old was 31.4% in Chiapas (*vs.* 13.6% in all Mexico), and a relationship between poverty, undernutrition and TB deaths has been reported for Chiapas [5, 24, 25]. In another study conducted between 2000 and 2009, Chiapas was the Mexican state with the highest mortality rate among TB patients, a finding that was associated with its low socio-economic classification [2].

The presence of indigenous communities and migrants are relevant to TB control in Chiapas given its high indigenous presence and border location with Guatemala. Indigenous populations bear a disproportionately high burden of TB worldwide despite the underdiagnoses of TB in these communities [26–30]. Certain indigenous populations appear to be genetically susceptible to type-2 DM, which can further increase TB risk [31]. Migrants are a key affected population by TB, and the WHO has guidelines to control TB among immigrants [32, 33]. In low incidence countries, 35–70% of the TB patients are foreign-born [34]. The combination of migration with DM or with indigenous origin magnifies the risk of TB [35].

We conducted a cross-sectional assessment of the prevalence of the most commonly reported risk factors for TB in Chiapas using TB surveillance data collected between 2010 and 2014. These included DM, undernutrition, HIV and excess alcohol consumption. We further evaluated their association with socio-demographic factors such as immigration or indigenous presence, and with unfavourable TB treatment outcomes, including death.

## Methods

### Data source

We conducted a secondary data analysis on all the TB cases documented by the Ministry of Health (Secretaría de Salud) in the state of Chiapas between 2010 and 2014. Details on the data collection process in Mexico are explained elsewhere [17]. Newly diagnosed TB patients are entered as ‘new’ to this database, and they begin the standard 6-month Directly Observed Therapy, Short course (DOTS) protocol per Mexican guidelines [36]. If a new treatment plan is initiated due to treatment failure, lost to follow-up or relapse, the same TB patient is re-entered into the database. To focus on the epidemiology of newly diagnosed TB patients and avoid duplicates, we only selected the ‘new TB episodes’. In this dataset, TB cases were confirmed by positive smears for acid-fast bacilli (AFB; 83.39%), histopathology (3%) or culture (0.31%). Patients with any form of TB outside the lungs were classified with extra-pulmonary, although some (e.g. miliary or mixed) also had pulmonary involvement. TB treatment outcomes were defined as: (i) ‘cured’ (complete treatment, no TB signs or symptoms by the end of it, and when available, at least two negative AFB smears or cultures), (ii) treatment failure (positive AFB at the end of treatment), (iii) ‘default’ (treatment interruption for ⩾30 days) or (iv) ‘death’ (from any cause during treatment).

### Selection of TB cases

**Fig. 1.**
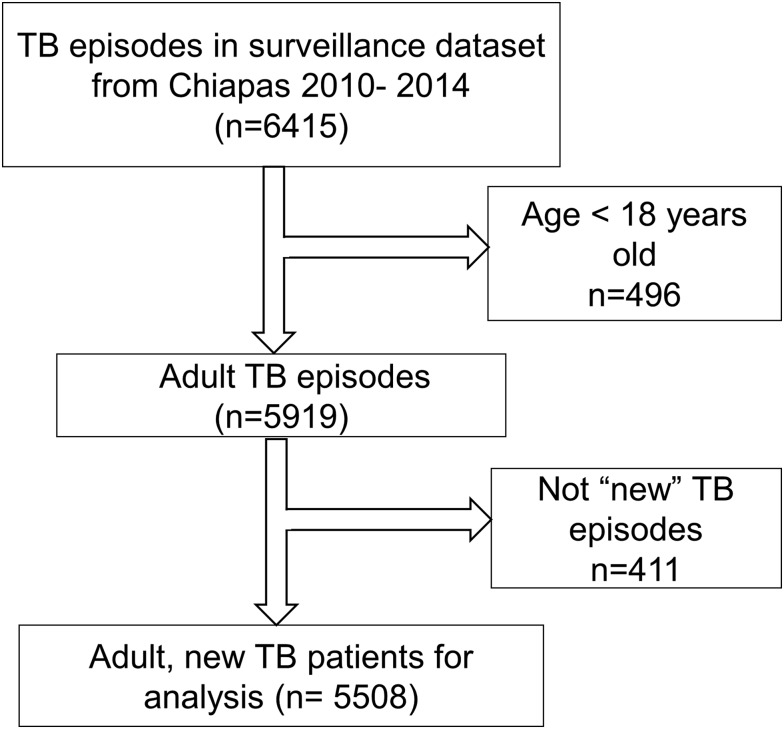
Selection of TB cases for data analysis. The initial sample consisted of 6415 records and final analysis was conducted on 5508. We first excluded TB episodes that did not meet the inclusion criteria of age (*n* = 496), and then excluded an additional 411 episodes that did not correspond to newly diagnosed cases (i.e. entered as treatment failure, relapse, referred or re-entry).

The initial dataset from Chiapas contained 6415 TB episodes reported between 2010 and 2014. We limited the analysis to 5508 newly diagnosed, adult TB patients (18 or older; Fig. 1). This study was reviewed and approved by The Committee for Protection of Human Subjects at the University of Texas-Houston, the research ethics committees from the Secretaría de Salud de Chiapas and El Colegio de la Frontera Sur (ECOSUR).

### Definitions

We collectively refer to ‘comorbidities’ as any listed concurrent diseases (e.g. DM, HIV, undernutrition), health conditions (e.g. pregnancy) or known social risk factors for TB (e.g. alcohol excess). The diagnosis of DM was based on capillary blood glucose testing (random or fasting) or self-reporting. HIV was based on two positive enzyme-linked immunosorbent assays with confirmation by Western blot. Undernutrition was defined as a binary variable based on a BMI cut-off of 18.5 kg/m^2^ but further stratification was not available. Excessive alcohol use was self-reported. Employment was analysed by grouping into four categories as a proxy for socio-economic status: (i) unemployed or jail inmates, (ii) agricultural work, (iii) retired or housewives and (iv) non-agricultural work or student (Table 1). The state of Chiapas is divided into 10 sanitary jurisdictions. Those bordering Guatemala include Tapachula, Comitán, Motozintla, Ocosingo and Palenque, and the non-border jurisdictions include Pichucalco, San Cristóbal de Las Casas, Tonalá, Tuxtla Gutiérrez and Villaflores. Chiapas is further divided into 122 municipalities and it is one of the Mexican states with recognised presence of indigenous people, comprising 28% of its population [37, 38]. Indigenous people are defined by the Mexican Constitution as people who are descendants from populations that inhabited Mexico before European colonisation and who still preserve their social, economic, cultural and political institutions, or part of them, and by self-adscription. As they have major presence in certain areas within the state, we categorised the municipalities of Chiapas into four groups, according to the percentage of indigenous people, as: (i) predominant, ⩾70%; (ii) moderate, 40–69.9%; (iii) low, 10–39.9% and (iv) very low, <10% of the population [38]. This classification is based on the percentage of people who speak an indigenous language as part of their preserved culture, and may be an underestimate of the proportions of people who identify themselves as indigenous, but do not speak their maternal language.

**Table 1.** Characteristics of TB patients in Chiapas-Mexico, 2010–2014

Variable	*n* = 5508	Proportion (%) (95% CI)^a^
Age groups (years)		
18–40	2617/5508	47.5 (46.2–48.8)
41–64	2102/5508	38.2 (36.9–39.4)
⩾65	789/5508	14.3 (13.4–15.2)
Sex		
Male	3198/5508	58.1 (56.8–59.4)
Female	2310/5508	41.9 (40.6–43.2)
Occupation categories		
Healthcare workers	47/5279	0.9 (0.6–1.1)
Jail inmates	40/5279	0.8 (0.5–1.0)
Work labour	2017/5279	38.2 (36.9–39.5)
Managerial work	674/5279	12.8 (11.9–13.7)
Student	164/5279	3.1 (2.6–3.6)
Retired	77/5279	1.5 (1.1–1.8)
Unemployed or homemakers	2260/5279	42.8 (41.5–44.1)
Occupation as proxy for SES		
Unemployed or jail inmates	428/5281	8.1 (7.4–8.8)
Agriculture work	1295/5281	24.5 (23.4–25.7)
Retired or housewives	1949/5281	36.9 (35.6–38.2)
Non-agriculture work or student ^b^	1609/5281	30.5 (29.2–31.7)
Born in Chiapas		
Yes	5413/5508	98.3 (97.9–98.6)
No	95/5508	1.7 (1.4–2.1)
Jurisdictions bordering Guatemala		
Border	3199/5508	58.1 (56.8–59.4)
Non-border	2309/5508	41.9 (40.6–43.2)
Municipalities by indigenous people		
Predominant (>70%)	551/5508	10.0 (9.2–10.8)
Moderate (40% to 69%)	575/5508	10.4 (9.6–11.2)
Low (10% to 39%)	434/5508	7.9 (7.2–8.6)
Very low (<10%)	3948/5508	71.7 (70.5–72.9)
Location of TB		
Pulmonary	4954/5508	89.9 (89.1–90.7)
Extra-pulmonary^c^	554/5508	10.1 (9.3–10.9)
Treatment Outcomes		
Cured	4013/5482	72.9 (72.0–74.4)
Completed	560/5482	10.2 (9.4–11.0)
Treatment failure	70/5482	1.3 (1.0–1.6)
Death	300/5482	5.5 (4.9–6.1)
Treatment default	256/5482	4.7 (4.1–5.2)
Transfer to another unit	18/5482	0.3 (0.2–0.5)
Undergoing treatment	265/5482	4.8 (4.3–5.4)
Comorbidities	2520/5508	45.8 (44.4–47.1)
Diabetes	1053/5508	19.1 (18.1–20.2)
Alcohol excess	281/5508	5.1 (4.5–5.7)
HIV	224/5508	4.1 (3.5–4.6)
Undernutrition	794/5508	14.4 (13.5–15.3)
Other	191/5508	3.5 (3.0–4.0)

a Column percentages based on the total number of cases with available information for each variable.

b This category includes non-agriculture work, managerial work, students and healthcare workers.

c Extra-pulmonary TB patients had lymph node TB (*n* = 117), genitourinary (*n* = 8), adrenal gland (*n* = 1), intestinal peritoneum (*n* = 25), meningeal (*n* = 12), eye (*n* = 1), bone (*n* = 40), other (*n* = 46), skin (*n* = 14), renal (*n* = 9), central nervous system (*n* = 1), miliary (*n* = 185), mixed (*n* = 38) or pleural TB (*n* = 57), with miliary and mixed also having pulmonary involvement; SES, socio-economic status.

### Statistical analysis

Descriptive statistics is provided. Some variables had missing information, notably *n* = 702 for comorbidities. Based on further examination of these missing data, for data analysis we assumed that the lack of comorbidity reporting indicated the absence of a comorbidity (Supplement and Table S1). Furthermore, we found that conclusions were not affected when alternative data analysis was conducted by excluding participants with missing comorbidity (data not shown).

We used a one sample test of proportions to compare prevalence of DM in TB patients with the general adult population of Chiapas. We used Pearson *χ*^2^ test for assessing possible association between two categorical variables, or Fisher's exact test when any cell size was <5. For continuous variables we used Student's *t* test when the variables were normally distributed, or its non-parametric counterpart, Wilcoxon signed-rank test, when the variables were not normally distributed. All multivariable logistic regression analyses were adjusted for age and sex. To increase statistical power of tests, comorbidities were merged into one variable for multivariable logistic regression model presented in Table 4. TB incidence was estimated based on the number of new TB patients in our database and the adult population size for each region based on census data for the corresponding years. For the nine participants who had more than one comorbidity, only one was selected with this order of priority: HIV (highest known risk for TB), undernutrition, DM, alcohol excess or others. Interactions between the independent variables were explored but none had biological significance and hence, are not shown. All analyses were performed using SAS software (SAS Institute, Cary, NC, version 9.4) at a significance level of 0.05.

## Results

### Characteristics of TB patients in Chiapas

#### Socio-demographics

We analysed data for 5508 new TB patients registered in Chiapas between 2010 and 2014. Their mean age was 43.7 years (s.d. 17.4 years) and nearly 60% were males (Table 1). The occupation group with the highest prevalence was the unemployed or homemakers (42.8%), followed by work labourers (38.2%). Nearly one-fourth was in agriculture work, which we used as a proxy for lower socio-economic status.

#### Geographic distribution of TB in Chiapas

**Fig. 2.**
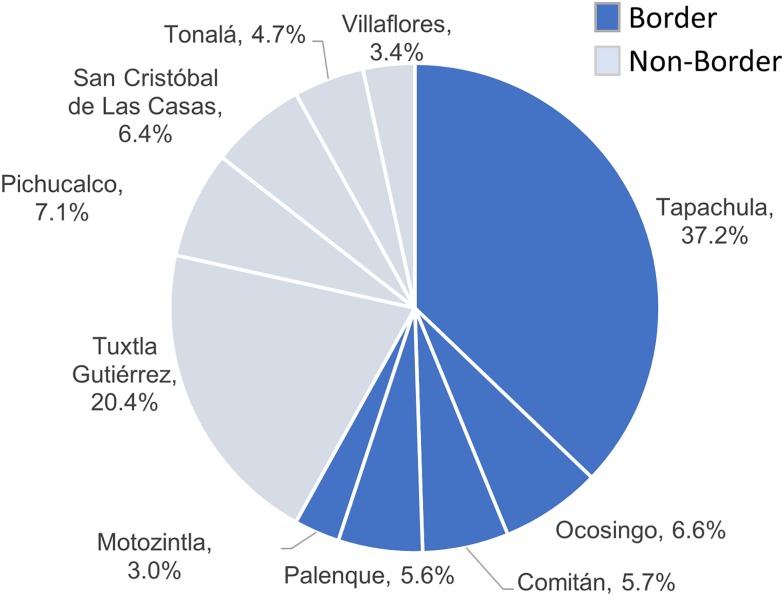
Distribution of TB cases in Chiapas by health jurisdiction and border location. Percent of total indicated for each health jurisdiction.

The southern region of Chiapas shares more than 620 km of border with Guatemala, and is a site of active migration. Even though a minority of the adult TB patients (1.7%) were not born in Chiapas, more than half of the TB cases in the state were from jurisdictions that have borders with Guatemala (58.1%), with most of these from Tapachula (37.2%; Fig. 2). Among the non-border jurisdictions, the highest proportion of TB cases was from Tuxtla Gutiérrez (20.4%), which is the capital city of Chiapas.

Chiapas has a high presence of indigenous communities, and these populations are at higher risk of TB [26]. However, we found that municipalities with predominant or moderate presence of indigenous people contributed to only 20.4% of all the TB cases in Chiapas. Likewise, the average 5-year incidence of TB was lower in municipalities with predominant indigenous presence (25/1 00 000/year) *vs.* very low presence (47.6/1 00 000/year).

#### TB characteristics

Pulmonary TB occurred in nearly 90% of the patients. TB treatment for all forms of TB was successful in 83.4% of the patients, and the most common unfavourable outcomes were treatment defaulting (4.7%) and death (5.5%; Table 1).

### TB comorbidities and their distribution by state geography and indigenous presence

**Fig. 3.**
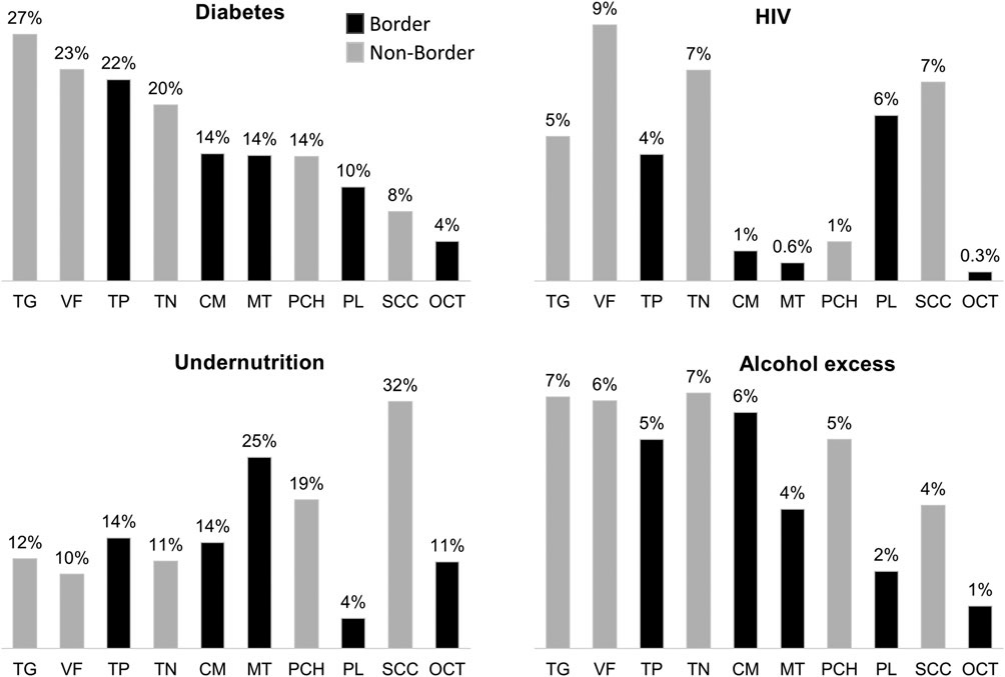
Distribution of DM and other comorbidities by sanitary jurisdictions of Chiapas. Border jurisdictions are in black, and non-border jurisdictions are in grey. TG, Tuxtla Gutierrez; VF, Villaflores; TP, Tapachula; TN, Tonala; CM, Comitan; MT, Motozintla; PCH, Pichucalco; PL, Palenque; SCC, San Cristobal de las Casas; OCT, Ocosingo.

Nearly half (45.8%) of the TB patients had at least one comorbidity. The most common were DM (19.1%), undernutrition (14.4%), excess consumption of alcohol (5.1%) and HIV (4.1%). Only nine participants (0.18%) reported more than one comorbidity. The frequency of each comorbidity was not similar throughout the state of Chiapas. When analysed by the 10 sanitary jurisdictions (Fig. 3), the proportion of DM ranged from 4.4% in Ocosingo, to 27.3% in Tuxtla Gutiérrez. The range was even wider for undernutrition where the lowest prevalence was 3.9% in Palenque, and the highest prevalence was 31.9% in San Cristóbal de Las Casas. Villaflores had the highest prevalence of HIV in TB patients (9.0%) and Ocosingo the lowest (0.3%), and Tonalá had the highest proportion of TB patients who reported excess consumption of alcohol (6.6%) and Ocosingo the lowest (1.0%).

**Fig. 4.**
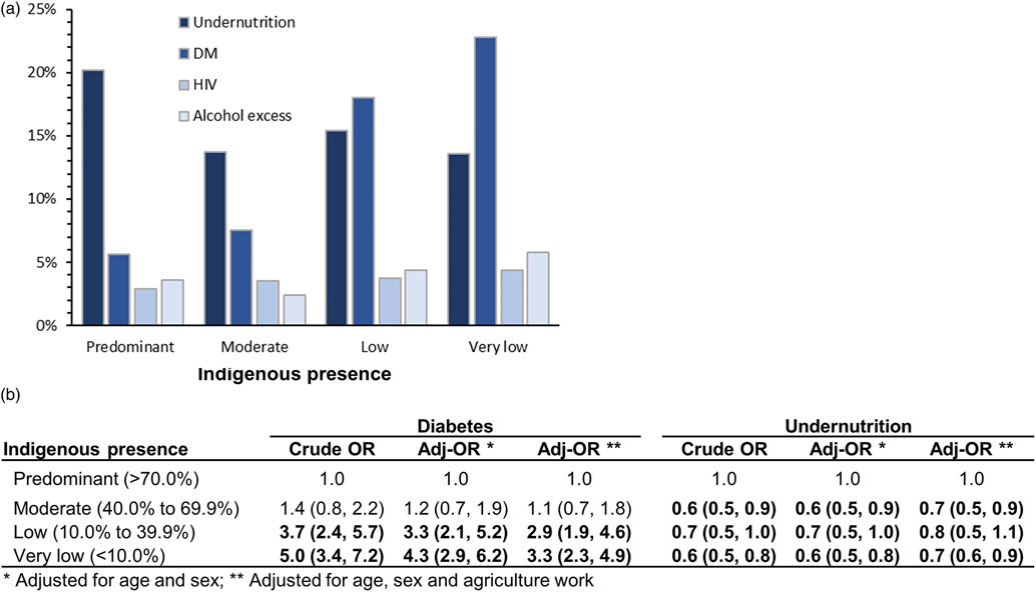
Distribution of comorbidities by indigenous presence. (a) Proportion of each comorbidity by indigenous presence. (b) Crude and adjusted OR of diabetes or undernutrition by indigenous presence.

The distribution of comorbidities differed based on the proportions of indigenous people. The most notable was that municipalities with predominant indigenous presence had the highest undernutrition (20.2%), followed by DM (5.6%), excessive consumption of alcohol (3.6%) and HIV (2.9%). Alternatively, the municipalities with the lowest indigenous presence had higher DM (22.8%), followed by undernutrition (13.6%), excessive consumption of alcohol (5.8%) and HIV (4.4%; Fig. 4). This differential distribution of DM and undernutrition was significant. Prevalence of DM was higher in municipalities with low (odds ratio (OR) 3.7, 95% confidence interval (CI) 2.4–5.7) or very low presence of indigenous people (OR 5.0, 95% CI 3.4–7.2; Fig. 4). This difference remained statistically significant after adjusting for age and sex, and after occupation as proxy for socio-economic status. Likewise, the distribution of undernutrition was also significantly associated with indigenous presence. Specifically, prevalence of undernutrition was about 0.6-fold (or 40% lower) in communities with moderate, low or very low indigenous presence, when compared with those with predominant indigenous presence (Fig. 4).

### DM was the most common comorbidity among TB patients

The average 5-year proportion of DM among TB patients (19.1%) was significantly higher than that of 5.6% DM in the general adult population of Chiapas in 2012, which relied on self-reported DM (*P* < 0.0001) [19]. When comparing socio-demographic characteristics, the patients with TB-DM (*vs.* TB-no DM) were more likely to be females, older than 40 years who had a non-agricultural occupation or were retired or housewives, and lived in non-border regions of the state (Table 2). After controlling for age and sex, similar findings were observed.

**Table 2.** Socio-demographic and medical characteristics in TB patients in Chiapas-Mexico by DM status

Variable	TB-DM (*n* = 1053)	TB-no DM^a^ (*n* = 4455)	Crude OR (95% CI)	Adjusted OR^b^ (95% CI)
Age groups				
18–40 years	174 (16.5%)	2443 (54.8%)	1.0	
41–64 years	717 (68.1%)	1385 (31.1%)	**7.3 (6.1–8.7)**	
⩾65 years	162 (15.4%)	627 (14.1%)	**3.6 (2.9–4.6)**	
Sex				
Male	537 (51.0%)	2661 (59.7%)	1.0	
Female	516 (49.0%)	1794 (40.3%)	**1.4 (1.2–1.6)**	
Occupation as proxy for SES				
Unemployed or jail inmates	59 (5.6%)	369 (8.3%)	1.0	1.0
Agriculture work	120 (11.4%)	1175 (26.4%)	**0.6 (0.5–0.9)**	**0.6 (0.4–0.9)**
Retired or housewives	477 (45.3%)	1472 (33.0%)	**2.0 (1.5–2.7)**	**2.4 (1.7–3.5)**
Non-agriculture work or student	360 (34.2%)	1249 (28.0%)	**1.8 (1.3–2.4)**	**2.8 (2.0–3.8)**
Born in Chiapas				
No	12 (1.1%)	83 (1.9%)	0.6 (0.3–1.1)	0.8 (0.4–1.4)
Yes	1041 (98.9%)	4372 (98.1%)	1.0	1.0
Jurisdictions bordering Guatemala				
Border	571 (54.2%)	2628 (59%)	1.0	1.0
Non-border	482 (45.8%)	1827 (41%)	**1.2 (1.1–1.4)**	**1.3 (1.1–1.5)**
Location of TB				
Extra-pulmonary	44 (4.2%)	510 (11.5%)	1.0	1.0
Pulmonary	1009 (95.8%)	3945 (88.6%)	**3.0 (2.2–4.1)**	**3.0 (2.2–4.1)**
Treatment outcomes				
Cured or completed	902 (85.7%)	3671 (82.4%)	1.0	1.0
Treatment failure	14 (1.3%)	56 (1.3%)	1.0 (0.6–1.8)	1.0 (0.61.9)
Death	45 (4.3%)	255 (5.7%)	0.7 (0.5–1.0)	**0.6 (0.4–0.8)**
Treatment default	40 (3.8%)	216 (4.9%)	0.8 (0.5–1.1)	0.8 (0.6–1.1)

a This is the reference group for all the analyses.

b Adjusted for age and sex.

Row proportions provided to illustrate the distribution of diabetes among each category; Statistically significant differences are highlighted with bold text; Others include: cirrhosis, intravenous drugs, acute pulmonary oedema, pregnancy, chronic obstructive pulmonary disease, heart failure, neoplasms, mixed and others.

Statistically significant differences are highlighted with bold text.

The TB-DM patients were more likely to present pulmonary TB (*vs.* extra-pulmonary TB) (OR 3.0, 95% CI 2.2–4.1) with respect to TB-no DM, and this difference remained significant after adjusting for age and sex (OR 3.0, 95% CI 2.2–4.1). The TB-DM patients were more likely to have successful TB treatment outcomes (with less treatment failure, death or defaulting of TB treatment). In fact, after adjusting for age and sex, TB patients with DM were 0.6-fold (or 40% less likely) of dying during TB treatment, compared with those without DM.

### Undernutrition – the second most prevalent comorbidity

Undernutrition affected 14.4% of the TB patients in Chiapas. It was more prevalent in communities with predominant indigenous presence (Fig. 4), in the young adults (18–40 years) and in those with occupations associated with lower socio-economic status (Table 3). When controlling for age and sex, similar characteristics remained associated with TB and undernutrition. TB patients with undernutrition were more likely to die during the course of TB treatment (OR 1.7, 95% CI 1.3–2.3) (Table 3).

**Table 3.** Socio-demographic and medical characteristics in TB patients in Chiapas-Mexico by undernutrition

Variable	TB- undernutrition (*n* = 794)	TB-no undernutrition^a^ (*n* = 4714)	Crude OR (95% CI)	Adjusted OR^b^ (95% CI)
Age groups				
18–40 years	444 (55.9%)	2173 (46.1%)	1.0	
41–64 years	210 (26.4%)	1892 (40.1%)	**0.5 (0.5–0.6)**	
⩾65 years	140 (17.6%)	649 (13.8%)	1.1 (0.9–1.3)	
Sex				
Male	445 (56.0%)	2753 (58.4%)	1.0	
Female	349 (44.0%)	1961 (41.6%)	1.1 (0.9–1.3)	
Occupation as proxy for SES				
Unemployed or jail inmates	76 (9.6%)	352 (7.5%)	1.0	1.0
Agriculture work	226 (28.5%)	1069 (22.7%)	1.0 (0.7–1.3)	1.0 (0.8–1.4)
Retired or housewives	291 (36.7%)	1658 (35.2%)	0.8 (0.7–1.1)	0.7 (0.5–1.0)
Non-agriculture work or student	173 (21.8%)	1436 (30.5%)	**0.6 (0.4–0.7)**	0.5 (0.4–0.7)
Born in Chiapas				
No	17 (2.1%)	78 (1.7%)	1.3 (0.8–2.2)	1.3 (0.7–2.2)
Yes	777 (97.9%)	4636 (98.4%)	1.0	1.0
Jurisdictions bordering Guatemala				
Border	430 (54.2%)	2769 (58.7%)	1.0	1.0
Non-border	364 (45.8%)	1945 (41.3%)	1.2 (1.0–1.4)	**1.2 (1.0–1.4)**
Location of TB				
Extra-pulmonary	70 (8.8%)	484 (10.3%)	1.0	1.0
Pulmonary	724 (91.2%)	4230 (89.7%)	1.2 (0.9–1.5)	1.2 (0.9–1.6)
Treatment outcomes				
Cured or completed	641 (80.7%)	3932 (83.4%)	1.0	1.0
Treatment failure	12 (1.5%)	58 (1.2%)	1.3 (0.7–2.4)	1.3 (0.7–2.4)
Death	62 (7.8%)	238 (5.1%)	**1.6 (1.2–2.1)**	**1.7 (1.3–2.3)**
Treatment default	42 (5.3%)	214 (4.5%)	1.2 (0.9–1.7)	1.2 (0.9–1.7)

a This is the reference group for all the analyses.

b Adjusted for age and sex.

Row proportions provided to illustrate the distribution of undernutrition among each category. Statistically significant differences are highlighted with bold text.

### TB treatment outcomes by DM status and other host factors

More than 10% of the TB patients reported one of three unfavourable TB treatment outcomes: treatment failure, death or default (Table 1). We evaluated the host factors associated with these unfavourable treatment outcomes, in addition to DM (Table 2) or undernutrition (Table 3). We provide the descriptive statistics for each type of treatment outcomes in Table 4, but for data analysis we merged the three categories into one (unfavourable outcomes) due to the limited sample sizes for some of these. When compared with patients with favourable outcomes (cured or completed treatment), those with unfavourable outcomes were more likely to be 65 years or older (OR 1.7, 95% CI 1.4–2.2), males (OR 1.6, 95% CI 1.4–1.9), unemployed or jail inmates (OR 1.6, 95% CI 1.2–2.1), immigrants (not born in Chiapas; OR 3.4, 95% CI 3.4 (2.2–5.4)) and to have extra-pulmonary TB (OR 2.2, 95% CI 1.7–2.8).

**Table 4.** Host characteristics predictive for increased odds of any unfavourable treatment outcome in TB patients; Chiapas-Mexico (*n*), 2010–2014^a^

Variable	Treatment failure (*n* = 70)	Death (*n* = 300)	Treatment default (*n* = 256)	Unfavourable outcome (*n* = 626)	Unfavourable outcome, crude OR	Unfavourable outcome, adjusted OR^b^
Age groups						
18–40 years	34 (1.3%)	101 (3.9%)	130 (5.0%)	265 (10.1%)	1.0	
41–64 years	24 (1.1%)	116 (5.5%)	90 (4.3%)	230 (10.9%)	1.1 (0.9–1.3)	
⩾65 years	12 (1.5%)	83 (10.5%)	36 (4.6%)	131 (16.6%)	**1.7 (1.4–2.2)**	
Sex						
Male	48 (1.5%)	198 (6.2%)	180 (5.6%)	426 (13.3%)	**1.6 (1.4–1.9)**	
Female	22 (1.0%)	102 (4.4%)	76 (3.3%)	200 (8.7%)	1.0	
Occupation as proxy for SES						
Agriculture work	20 (1.5%)	82 (6.3%)	64 (4.9%)	166 (12.8%)	1.0	1.0
Non-agriculture or student	21 (1.3%)	66 (4.1%)	81 (5.0%)	168 (10.4%)	0.8 (0.6–1.0)	1.0 (0.8–1.2)
Retired or housewives	19 (1.0%)	95 (4.9%)	64 (3.3%)	178 (9.1%)	**0.7 (0.6–0.9)**	1.1 (0.8–1.6)
Unemployed or jail inmates	8 (1.9%)	39 (9.1%)	32 (7.5%)	79 (18.5%)	**1.6 (1.2–2.1)**	**1.7 (1.2–2.2)**
Born in Chiapas						
No	5 (5.3%)	6 (6.3%)	17 (17.9%)	28 (29.5%)	**3.4 (2.2–5.4)**	**3.5 (2.2–5.6)**
Yes	65 (1.2%)	294 (5.4%)	239 (4.4%)	598 (11.1%)	1.0	1.0
Jurisdictions bordering Guatemala						
Border	43 (1.3%)	158 (4.9%)	162 (5.1%)	363 (11.4%)	1.0	1.0
Non-border	27 (1.2%)	142 (6.2%)	94 (4.1%)	263 (11.4%)	1.0 (0.8–1.2)	1.0 (0.9–1.2)
Municipalities by indigenous people^c^						
Predominant	3 (0.5%)	32 (5.8%)	23 (4.2%)	58 (10.5%)	1.0	1.0
Moderate	1 (0.2%)	28 (4.9%)	21 (3.7%)	50 (8.7%)	0.8 (0.5–1.2)	0.8 (0.5–1.1)
Low	11 (2.5%)	21 (4.8%)	16 (3.7%)	48 (11.1%)	1.1 (0.7–1.6)	1.0 (0.7–1.5)
Very low	55 (1.4%)	219 (5.6%)	196 (5.0%)	470 (11.9%)	1.1 (0.8–1.5)	1.1 (0.8–1.4)
Comorbidities^d^						
DM	14 (1.3%)	45 (4.3%)	40 (3.8%)	99 (9.4%)	1.0 (0.8–1.3)	1.0 (0.7–1.2)
Alcohol excess	4 (1.4%)	14 (5.0%)	22 (7.8%)	40 (14.2%)	**1.7 (1.2–2.4)**	**1.4 (1.0–2.0)**
HIV	2 (0.9%)	54 (24.1%)	12 (5.4%)	68 (30.4%)	**4.8 (3.5–6.5)**	**5.0 (3.6–6.9)**
Undernutrition	12 (1.5%)	62 (7.8%)	42 (5.3%)	116 (14.6%)	**1.7 (1.4–2.2)**	**1.7 (1.4–2.2)**
Others	3 (1.6%)	21 (11.0%)	10 (5.2%)	34 (17.8%)	**2.5 (1.7–3.7)**	**2.4 (1.6–3.6)**
Among DM, years with DM (median, inter-quartile range (IQR))^e^						
TB location	3.8 (6.7)	9.4 (15.1)	6.6 (9.1)	6.7 (11.1)	1.0 (1.0–1.1)	1.0 (1.0–1.1)
Extra-pulmonary	0 (0.0%)	62 (11.2%)	32 (5.8%)	94 (17.0%)	**2.2 (1.7–2.8)**	**2.4 (1.9–3.1)**
Pulmonary	70 (1.4%)	238 (4.8%)	224 (4.5%)	532 (10.7%)	1.0	1.0

a Row proportions are provided to illustrate the distribution of DM among TB patients in each category.

b OR adjusted for age and sex for all variables.

c Predominant (>70%), moderate (40% to 69%), low (10% to 39%), very low (<10.0%). Numbers in bold are significant.

d Comorbidities were merged into one variable that was used in the multivariable logistic regression model. This is to control for the effect of the rest of the comorbidities when estimating the association between DM and TB treatment outcome.

e Reported as median (IQR) due to moderate to severe skewness within study groups.

To evaluate the contribution of the most common comorbidities (DM, alcohol excess, undernutrition, HIV and others) to unfavourable TB treatment outcomes, we merged all the comorbidities into one variable. In the univariate logistic regression analysis, alcohol excess, undernutrition and HIV increased the odds of unfavourable events, but DM did not. After adjusting for age and sex, DM continued to have no impact and alcohol excess was no longer associated with unfavourable outcomes. However, unfavourable outcomes were still more likely among TB patients with undernutrition (adj-OR 1.7, 95% CI 1.4–2.2), other comorbidities (adj-OR 2.4, 95% CI 1.6–3.6) and HIV (adj-OR 5.0, 95% CI 3.6–6.9). Further analysis among those with DM only, also showed that there was no difference in the median number of years with DM awareness and odds of unfavourable treatment TB outcomes.

## Discussion

We analysed the epidemiology of the most common comorbidities for TB using the official surveillance data from the state of Chiapas. Among the Mexican states, Chiapas has a strong indigenous presence (27%), is the poorest and shares a highly mobile border with Guatemala [3, 37]. Chiapas is ranked 11th among the 32 Mexican states for TB incidence, and 6th in TB mortality (2.7/100 000) [4]. However, Chiapas is known for its chronic under-reporting of all TB cases, and its nationwide ranking is likely to be higher [5, 27].

DM was the most prevalent comorbidity among TB patients in Chiapas, affecting one-fifth of the TB patients. This 19% prevalence is among the highest in the world [39, 40] and is similar to previous reports from Mexico based on passive reporting (range 17.8–25.2%) [9, 17, 41], but lower than other Mexican studies using active TB surveillance (range 29.6–36%) [7, 8, 16]. Mexico implemented routine blood testing for diabetes in 2010 for all newly diagnosed adult TB patients, but we anticipate that our estimates are under-reported given the notable increase in DM prevalence between 2010 (17.4%) and 2014 (21.6%) in this study. Over time the routine implementation of the programme and associated data entry is expected to improve. Accordingly, in 2017 the reported TB-DM prevalence was 29.8% (Castellanos-Joya, unpublished findings from the Ministry of Health). These findings reinforce the importance of implementing an integrated management of TB and DM in Chiapas, after the pilot testing done in recent years in Mexico [42].

The patients with TB-DM had distinguished characteristics. They were older females and more likely to present with pulmonary (*vs.* extra-pulmonary) TB. They were less likely to have HIV, undernutrition or report excessive alcohol consumption. These findings are consistent with previous reports on the unique characteristics of TB-DM *vs.* TB-no DM [7, 9, 17, 41]. However, in Chiapas we found that the TB-DM patients appeared to have a higher socio-economic status when compared with TB-no DM. This finding differs from previous observations in Mexico [9, 17], and can be explained by the higher poverty levels in Chiapas compared with other Mexican states, where undernutrition among the TB-no DM patients is prevalent (21%). This contrasted with the prevalence of DM in Chiapas, which was higher in the more affluent regions of the state, like Tuxtla Gutiérrez or Villaflores. The contrasting epidemiology between DM and undernutrition reflects the need for different approaches to TB control programmes in distinct regions of the state. These two comorbidities combined add up to 33.5% of the TB patients, indicating the fundamental contribution of metabolic alterations to TB risk – this deserves further study.

Border communities are likely to have more migrant populations in search of better job opportunities or escaping from areas of conflict. Migrants worldwide usually settle in unhealthy environments that favour TB development [28]. The exposure to social, economic, and biological risk factors for TB is an intrinsic part of the migration process [32, 43]. In Chiapas, the sanitary jurisdiction of Tapachula has refugee shelters and is the main passage for migrants from Central America (Guatemala, Honduras, El Salvador), the Caribbean (Haiti, Cuba), South America (Ecuador, Brazil) and other countries [28, 44]. Accordingly, Tapachula had the highest proportion of immigrant TB patients (92% of the 1.7% who were born outside of Chiapas). Notably, immigrant TB patients were more likely to have unfavourable TB treatment outcomes, with the most prevalent being treatment defaulting in 18% (*vs.* 4% of the Chiapas-born residents; Table 4). In addition to migration, the border sanitary jurisdiction of Tapachula also has more HIV and a reference hospital for management of MDR-TB.

We found that deaths were more likely among TB patients with HIV or undernutrition, which is consistent with the literature [22, 23, 45]. In contrast, DM was not associated with higher deaths, a finding consistent with our previous studies in Mexico, but not with reports from other populations [9, 46–48]. The absence of an association between death and DM in our study may have several explanations. First, deaths in TB-DM patients may be more frequent when there are additional comorbidities that synergise to trigger mortality. There may be a variable distribution between study populations worldwide on the co-occurrence of DM with other risk factors for TB deaths (e.g. HIV, undernutrition, poverty, access to healthcare). Our results may be explained by the absence of HIV or undernutrition in DM patients, two risk factors strongly associated with death. Second, TB-DM patients may receive more rigorous medical monitoring due to their DM status when compared with non-DM. Third, it has been suggested that the higher death in TB-DM patients is not due to TB, but to DM complications such as cardiovascular diseases [49, 50]. Accordingly, in a recent principal component analysis we found that cardiovascular risk factors (low density cholesterol, triglycerides and central obesity) were correlated with factors defining DM (haemoglobin A1c (HbA1c) and insulin resistance) in participants from South Africa, but not in Mexican Hispanics [51]. Finally, in our study a proportion of the TB patients (4.7%) were not followed when they defaulted treatment, and we were not able to assess other poor outcomes such as TB recurrences and relapses, which are reportedly associated with DM [13, 52].

The indigenous communities in Mexico, Chiapas included, live in difficult conditions that favour TB, such as poverty and being marginalised [28, 29, 53]. This is consistent with the higher burden of TB among indigenous people worldwide [26]. Thus, one would expect that TB would be more prevalent in municipalities where indigenous people are predominant, but we found the opposite: lower prevalence and incidence of TB in communities with higher proportions of indigenous people. A possible explanation for our unexpected finding is that Tapachula, the jurisdiction with the highest TB prevalence, has a low proportion of indigenous people. Their higher numbers of TB likely stem from other factors. Namely, Tapachula is a major development hub in Chiapas, with the highest budget for TB management and hence, higher likelihood for better TB reporting and monitoring. It also has the highest prevalence of HIV/AIDS (together with Tuxtla-Gutierrez). In 2015 it occupied the ninth highest place for number of TB cases in Mexico [54, 55]. Given these unique characteristics, we excluded Tapachula and re-calculated the incidence of TB among the four groups of municipalities classified by indigenous presence. However, results did not differ after excluding Tapachula. Another plausible explanation is the possible under-reporting of TB in the municipalities with predominant indigenous presence. Several studies carried out in different regions of Chiapas, have demonstrated high levels of under-diagnosis of TB in indigenous communities [27–29] with only 45% of their cases being detected and reported by officials. Accordingly, another study found that being indigenous was a protective factor for MDR-TB, but their disproportionate under-diagnosis (*vs.* non-indigenous) is a likely source of error for this conclusion [56].

Our study has the limitations inherent to a secondary data analysis. In addition to the possible under-reporting of DM, there was no information on glucose control (HbA1c), which has been most strongly associated with TB risk and immunological alterations [39, 57]. The dataset also lacked information on the clinical characteristics of the TB patients, including smear status, cavitary or miliary TB, which are independent risk factors for poor TB treatment outcomes. Furthermore, we did not have quantitative data to further characterise the state of undernutrition of the TB patients, and when present, we could not distinguish if undernutrition was a cause or consequence of TB. Also, we cannot determine if the cause of death was TB or another comorbidity. Drug resistance was not evaluated due to missing data on a significant proportion of the cases, despite the high prevalence of MDR-TB in Chiapas [56]. We acknowledge that several variables had more than 10% of missing data, but our imputation analysis suggested the potential presence of non-differential misclassification and hence, did not affect the conclusions of our study. Finally, there is a likelihood of under-reporting of TB cases as discussed above. Missing TB case is a real threat to the effectiveness of the TB control programmes. Therefore, it is necessary to address this concern and assess the performance of TB surveillance programmes in these areas, and the obstacles it is facing.

## Supplementary material

For supplementary material accompanying this paper visit https://doi.org/10.1017/S0950268818003461. click here to view supplementary material
