# Supplementary material for: Diabetes, undernutrition, migration and indigenous communities: tuberculosis in Chiapas, Mexico
Source: Epidemiol Infect. 2019 Jan 25;147:e71. doi: 10.1017/S0950268818003461 (PMC6518577; doi:10.1017/S0950268818003461)
Supplement: Supplementary file 1 [file S0950268818003461sup001.docx]

**Epidemiology and Infection**

Diabetes, undernutrition, migration and indigenous communities: Tuberculosis in Chiapas, Mexico

H. A. RASHAK^1^, H. J. SÁNCHEZ-PÉREZ^2^, B. E. ABDELBARY^3^, A. BENCOMO-ALERM^4^ N. ENRIQUEZ-RÍOS^5^, A. GÓMEZ-VELASCO^2^, A. COLORADO^6^, M. CASTELLANOS-JOYA^7^, M. H. RAHBAR^8^, B. I. RESTREPO^1^*

**Supplementary Material**

**Analysis of missing data for TB comorbidities.**

Our focus for this paper was TB comorbidities and due to the large number of participants missing this information (n=702, 13% missing), we first verified with the health officials in Mexico. The health officials considered that leaving the comorbidity fields empty is most likely to occur when the comorbidity is not reported. This is consistent with the lower reporting of comorbidities in younger patients (expected to have less comorbidities). Based on this information we conducted our main analysis coding the missing comorbidities as zero or no comorbidity.

Futhermore, we evaluated if the data was missing at random or in particular participants. We found a significantly higher proportion of missing data on comorbidities for TB patients who were 18-40 years old (p=0.02), in agriculture work (p <0.001), in non-border jurisdictions, in municipalities with predominant, moderate or low (versus very low) indigenous presence (p <0.001) and in the year 2012 (p <0.001) (data not shown). Finally we repeated our analysis on how comorbities could be a factor in overestimating the odds of adverse outcomes leaving those missing observations as missing (tables S1) and using the multiple imputation method by chained equations method (MICE; Table S2). In table S1, the odds of any adverse outcome was similar to that found in table 4, with minor CI differences observed but otherwise no change from our main analysis. In table S2, similar results were found except for HIV where the odds of adverse outcome decreased to 2.3 (1.7, 3.1) both in the unadjusted and adjusted models. In addition, when evaluating the variable’s variance, the unadjusted model showed a higher estimated relative increase in variance for HIV (28.5 %) than expected had the data been complete. The increased in variance due to the missing information on comorbidities led to 13.3% increase in the standard error for the HIV comorbidity in the unadjusted model and 13.6% for the adjusted model. For other comorbidities the estimated relative increase in variance ranged from 0.8 % - 2.5% with an increase in the standard error ranging from 0.4 – 1.2 %. In conclusion the increase in variance for all comorbidities was negligible eliminating the possibility of biased estimation in our main analysis except for HIV. The results of the multiple imputation analysis warrants caution when interpreting the odds of adverse outcomes for those TB patients with concomitant HIV.

**Table S1: Comorbidities predictive for increased odds of any adverse treatment outcome in TB patients; Chiapas-Mexico (n), 2010-2014* (comorbidities including missing as missing)**

| Variable | Treatment Failure  N = 70 | Death  N = 300 | Treatment Default  N = 256 | Adverse outcome  n= 626 | Adverse outcome  Crude OR | Adverse outcome,  Adjusted OR^**^ |
| --- | --- | --- | --- | --- | --- | --- |
| Comorbidities***  No comorbidities  DM  Alcohol excess  HIV  Undernutrition  Others  Missing | 31 (1.4%)  14 (1.4%)  4 (1.5%)  2 (0.9%)  12 (1.6%)  3 (1.8%)  4 (0.6%) | 71 (3.3%)  45 (4.5%)  14 (5.0%)  54 (26.6%)  62 (8.2%)  21 (12.9%)  34 (5.1%) | 95 (4.4%)  40 (4.0%)  22 (8.4%)  12 (5.9%)  42 (5.6%)  10 (6.1%)  35 (5.3%) | 197 (9.1%)  99 (9.9%)  40 (14.9%)  68 (33.5%)  116 (15.3%)  34 (20.9%)  73 (11.0%) | 1  1.1 (0.8, 1.4)  **1.7 (1.2, 2.5)**  **5.0 (3.6, 6.9)**  **1.8 (1.4, 2.3)**  **2.6 (1.7, 3.9)**  1.2 (0.9, 1.6) | 1  1.0 (0.8, 1.3)  **1.5 (1.0, 2.1)**  **5.2 (3.7, 7.3)**  **1.8 (1.4, 2.3)**  **2.5 (1.7, 3.8)**  1.2 (0.9, 1.6) |

*Row proportions are provided to illustrate the distribution of DM and other comorbidities among TB patients in each category. **OR adjusted for age and sex. *** Comorbidities were merged into one variable that was used in the multivariable logistic regression model. This is to control for the effect of the rest of the comorbidities when estimating the association between DM and TB treatment outcome.

**Table S2: Comorbidities predictive for increased odds of any adverse treatment outcome in TB patients; Chiapas-Mexico (n), 2010-2014 using MICE procedure**

| Variable | Adverse outcome  Crude OR | Adverse outcome,  Adjusted OR^*^ |
| --- | --- | --- |
| Comorbidities**  No comorbidities  DM  Alcohol excess  HIV  Undernutrition  Others | 1  1.1 (0.8, 1.4)  **1.7 (1.2, 2.5)**  **2.3 (1.7, 3.1)**  **1.8 (1.4, 2.2)**  **2.5 (1.7, 3.8)** | 1  0.98 (0.8, 1.3)  **1.4 (1.0, 2.1)**  **2.3 (1.7, 3.1)**  **1.8 (1.4, 2.2)**  **2.4 (1.6, 3.7)** |

*OR adjusted for age and sex. ** Comorbidities were merged into one variable that was used in the multivariable logistic regression model. This is to control for the effect of the rest of the comorbidities when estimating the association between DM and TB treatment outcome.
